# The effect of Trolox on the rabbit anal sphincterotomy repair

**DOI:** 10.1186/s12876-023-02842-z

**Published:** 2023-06-20

**Authors:** Arash Sarveazad, Abazar Yari, Farnad Imani, Farimah Fayyaz, Marjan Mokhtare, Arash Babaei-Ghazani, Mahmoud Yousefifard, Shahriar Sarveazad, Siavash Assar, Jebreil Shamseddin, Mansour Bahardoust

**Affiliations:** 1 Colorectal Research Center, Iran University of Medical Sciences, Tehran, Iran; 2 Nursing Care Research Center, Iran University of Medical Sciences, Tehran, Iran; 3 Department of Anatomy, Faculty of Medicine, Alborz University of Medical Sciences, Karaj, Iran; 4 Pain Research Center, Department of Anesthesiology and Pain Medicine, Iran University of Medical Sciences, Tehran, Iran; 5 Neuromusculoskeletal Research Center, Department of Physical Medicine and Rehabilitation, Iran University of Medical Sciences, Tehran, Iran; 6 Department of Physical Medicine and Rehabilitation, University of Montreal Health Center, Montreal, Canada; 7 Physiology Research Center, Iran University of Medical Sciences, Tehran, Iran; 8 Department of Anesthesiology, kerman university of medical sciences, kerman, Iran; 9 Infectious and Tropical Diseases Research Center, Hormozgan Health Institute, Hormozgan University of Medical Sciences, Bandar Abbas, Shahid Chamran Boulevard Iran; 10 Department of Epidemiology, School of Public Health, Shahid Beheshti University of Medical Sciences, Velenjak 7th Floor, Bldg No.2 SBUMS, Arabi Ave, Tehran, 19839-63113 Iran

**Keywords:** Trolox, Fecal incontinence, External anal sphincter, Rabbit

## Abstract

**Introduction:**

Fecal incontinence (FI) is caused by external anal sphincter injury. Vitamin E is a potential strategy for anal sphincter muscle repair via its antioxidant, anti-inflammatory, anti-fibrotic, and protective properties against myocyte loss. Thus, we aimed to evaluate the water-soluble form of vitamin E efficacy in repairing anal sphincter muscle defects in rabbits.

**Methods:**

Twenty-one male rabbits were equally assigned to the intact (without any intervention), control (sphincterotomy), and Trolox (sphincterotomy + Trolox administration) groups. Ninety days after sphincterotomy, the resting and squeeze pressures were evaluated by manometry, and the number of motor units in the sphincterotomy site was calculated by electromyography. Also, the amount of muscle and collagen in the injury site was investigated by Mallory’s trichrome staining.

**Results:**

Ninety days after the intervention, the resting and squeeze pressures in the intact and Trolox groups were significantly higher than in the control group (P = 0.001). Moreover, the total collagen percentage of the sphincterotomy site was significantly lower in the Trolox group than in the control group (P = 0.002), and the total muscle percentage was significantly higher in the Trolox group compared to the control group (P = 0.001). Also, the motor unit number was higher in the Trolox group than in the control group (P = 0.001).

**Conclusion:**

Trolox administration in the rabbit sphincterotomy model can decrease the amount of collagen and increase muscle, leading to improved anal sphincter electromyography and manometry results. Therefore, Trolox is a potential treatment strategy for FI.

## Background

Pelvic floor disorders (PFDs), caused by weakened pelvic muscle, connective tissue, and fascia, are associated with decreased quality of life and increased economic burden. Impairment of defecation is a public health issue with a prevalence of approximately 37% in Iran [1–3]. Since fecal incontinence (FI) results from reduced mechanical pressure of anal sphincter tissue and the inability to obstruct the anal canal, anal sphincter injury or defect, as one of the PFDs, is considered among the leading causes of FI [4, 5]. Epidemiologic studies demonstrate that 2–15% of the population suffer from FI [6–8], and the prevalence increases to 13–23% with older age and in the female gender (possibly due to labor injuries) [9]. Although FI has enormous negative impacts on a patient’s daily life, social activities, quality of life, and mental health, it lacks a complete and standard treatment [6, 10, 11]. Although there are controversies about the effectiveness of surgery in the long term for the treatment of traumatic injuries of the anal sphincter in some cases [12], but still anal sphincter repair surgery is the primary treatment approach for anal sphincter injury [13]. Other treatment methods such as artificial anal sphincter and using mesh, are not ideal due to their high morbidity rates and the possibility of device failure [14, 15]. Efficacy of bulking agents is limited by various factors, such as absorbance of the injected agent, their migration, and fat emboli and granuloma formation [16, 17]. Therefore, there is increasing gravitation towards supplementary treatment approaches that restore the lost tissue to maintain efficacy in long-term follow-up.

Antioxidants, including Vitamin E, are among the agents that can repair the muscle tissue of the sphincter in anal sphincter injury, leading to the control of FI. Vitamin E is effective in repairing striated muscle (voluntary) through its antioxidant effect, inhibiting inflammatory factors, such as interleukin-6 (anti-inflammatory effect) [18, 19], its angiogenic effect [20], and protecting myocytes from membrane damage and loss [21]. Another property of vitamin E is its anti-fibrotic effect by decreasing TGF-β expression, which is why vitamin E could be helpful in anal sphincter muscle repair and controlling FI [22, 23].

Trolox (6-hydroxy-2 5 7 8-tetramethyl chroman-2-carboxylic acid), a water-soluble vitamin E, is commonly used in experimental studies since it is easily administered orally in laboratory animals due to its powder form and water-solubility [24]. Accordingly, vitamin E could be an efficient strategy for repairing muscles due to its antioxidant, anti-inflammatory, angiogenic, and anti-fibrotic properties and ability to prevent muscle cell loss. Thus, we aimed to evaluate the effect of vitamin E in repairing external anal sphincter (EAS) muscle defects in rabbits.

## Methods

### Study design

The present study was conducted after obtaining the approval of the ethical committee of the Iran university of medical sciences, and the “International Guiding Principles for Biomedical Research Involving Animals” guidelines established in 1985 were followed. The present experimental study was designed and performed according to the “Animal Research: Reporting of In Vivo Experiments” or ARRIVE guidelines. In this investigation, 21 male rabbits were divided into three groups (n = 7). To minimize the bias, the animals were randomly assigned to their group, and the investigators who performed the experiment and gathered and analyzed the data were blinded to the group allocation. Each rabbit was kept in a separate cage throughout the study procedure.

### Grouping

In the present study, 21 New Zealand white male rabbits (weighing between 2.5 and 3 kg provided by Pasteur Institute of Iran) were randomly assigned to three equal groups (n = 7):

1. Intact group: intact animals without any intervention.
2. Control group: animals undergoing sphincterotomy without treatment.
3. Trolox group: oral administration (gavage) of Trolox for 14 days (five days before and nine days after sphincterotomy) with a dosage of 40 mg/kg/day.

### Procedures and outcomes of the study

#### Sphincterotomy

The fourth-degree rupture sphincterotomy was performed based on the 1999 Sultan classification (EAS, internal anal sphincter (IAS), and anal mucosa tear) [25]. Similar to our previous study [26], following anesthesia using ketamine (80 mg/kg) and xylazine (10 mg/kg) in all rabbits, the anus and perianal area of the left side of the sphincter were disinfected, and sphincterotomy was performed in lithotomy position. The length of the lesion is 1 cm and the extension of the lesion is the thickness of a surgical blade bisturi No 11.

#### Trolox administration

Trolox was administered orally (gavage) for 14 days (five days before and nine days after sphincterotomy) with a dosage of 40 mg/kg/day [21, 22].

#### Resting and maximum squeeze pressure measurement (manometry)

The standard anorectal catheter (7.4 mm) and a pressure transducer (ON, L4Z 3L3, Mississauga, Canada) were used for manometry. Three months after sphincterotomy, the probe was inserted into the rectum, and the balloon (baseline pressure) was positioned to measure the resting and maximum squeeze pressures by pulling out the probe (constant rate of 0.05 cm/s). The pressure of the anal sphincter was recorded three times for each rabbit. Manometry of animals was performed without anesthesia and in full consciousness (no sedative was used).

#### Electromyography

Three months after sphincterotomy in order to record electromyography of the sphincterotomy site, Synergy on Nicolet EDX System (Natus Medical Corporate USA) disposable electrode and needle (Gauge 30, diameter of 0.3 mm, length of 25 mm, and recording area of 0.02 mm2/Ambu Copenhagen, Denmark) were used. Following lithotomy positioning (without anesthesia or relaxant), the electromyography needle was inserted vertically with a depth of 0.5 cm at the sphincterotomy site (at the junction of anal mucous and skin). After setting the system (Sweep:10ms/cm and Sensitivity:100 to 200), the number of motor unit action potentials (MUAPs) in 20 s was recorded for each rabbit. Electromyography of animals was performed without anesthesia and in full consciousness (no sedative was used).

#### Histopathology examination

Three months after sphincterotomy, rabbits were deeply anesthetized with ketamine (80 mg/kg) and xylazine (10 mg/kg), followed by transcardiac perfusion with 4% paraformaldehyde solution. The anal sphincter was entirely removed and fixed overnight with 4% paraformaldehyde. After tissue setting in paraffin, 10- µm transverse serials sections were prepared for histologic examination.

#### Mallory’s trichrome staining and quantitative assay of collagen

Three 10-µm sections (from inner, middle, and outer parts) were selected and stained by Mallory’s Trichrome in every rabbit. In the images of the sphincterotomy site under a light microscope x40, the total tissue area and the area occupied by Trichrome staining were measured by ImageJ/Fiji 1.46 software for each section. Ultimately, the ratio of the Trichrome stained area to the total tissue area was calculated and considered as the amount of collagen. The amount of muscle was calculated by subtracting the area occupied by Trichrome staining from the total tissue area [26, 27].

### Statistical analysis

Quantitative data were presented in mean (standard deviation). The non-parametric version of the ANOVA (Kruskal-Wallis) test was used to compare the findings between the three groups.

Data were analyzed using SPSS version 16.0 (SPSS Inc., IBM Company), and a P-value of less than 0.05 was considered statistically significant.

## Results

### Manometry

**Fig. 1:**
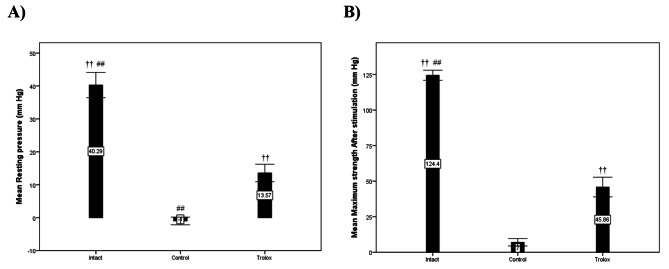
(**A**) Resting and (**B**) Maximum Strength After stimulation (squeeze) pressure in the study groups. Data are presented as mean ± SEM (n = 7 per group). †† Significant level at p-value < 0.0001 with the control group. ## Significant level at p-value < 0.0001 with the Trolox group

Manometry evaluated the sphincter contractility strength of 21 cases (seven cases for each group). The results demonstrated that the resting and maximum strength after stimulation (squeeze) pressures were significantly lower in the control and Trolox groups than in the intact group (p = 0.001). To be more specific, the resting pressure was significantly higher in the intact (40.29 ± 5.09) and Trolox (13.57 ± 3.50) groups than in the control group (-1.01 ± 1.52) (p = 0.001), and the maximum strength after stimulation (squeeze) pressure was significantly higher in the intact (124.43 ± 4.65) and Trolox (45.86 ± 9.15) groups compared to the control group (7.00 ± 3.49) (p = 0.001). The manometry results are presented in Table 1; Fig. 1.

**Table 1:** Resting and Maximum Strength After stimulation (squeeze) pressure in the study groups

Variable	Intact group (N = 7)	Control group (N = 7)	Trolox group (N = 7)	P-value
Resting pressure (mm Hg)	40.29 ± 5.09	-1.01 ± 1.52	13.57 ± 3.50	0.001
Maximum strength After stimulation (mm Hg)	124.43 ± 4.65	7 ± 3.49	45.86 ± 9.15	0.001

### Collagen and muscle tissue content

**Fig. 2:**
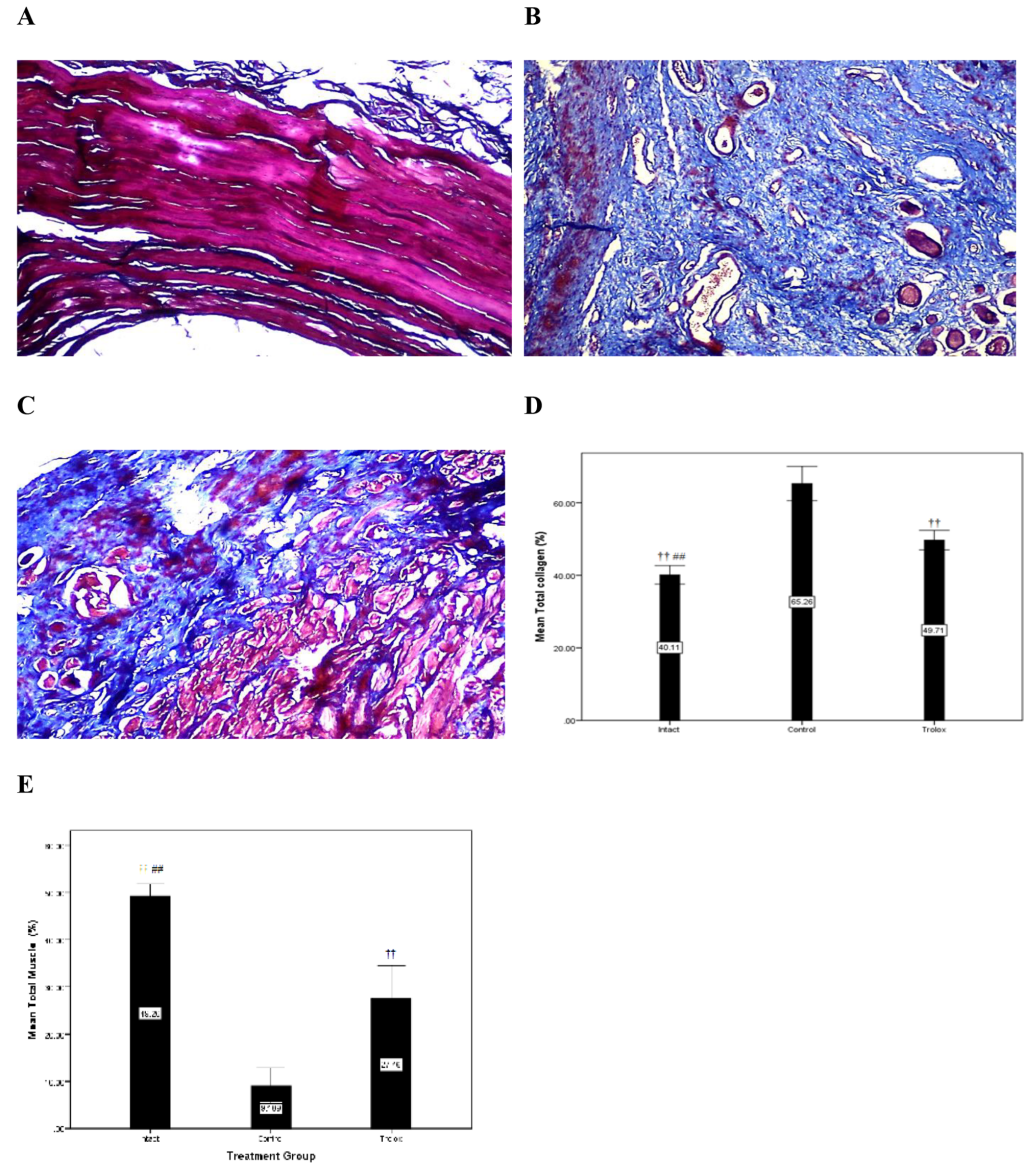
**Collagen and muscle amount in the study groups**. (**A**) Intact, (**B**) Control, and (**C**) Trolox group. (**D**) Percentage of collagen and (**E**) muscle. Data are presented as mean ± SEM (n = 7 per group). †† Significant level at p-value < 0.0001 with the control group. ## Significant level at p-value < 0.0001 with the Trolox group

Sixty-three tissue sections (three sections per each animal and a total of 21 sections for each group) were analyzed for the collagen and muscle content via Image J software. The total collagen ratio was significantly lower in the intact group (40.11 ± 3.8) than in the control (65.26 ± 7.05) and Trolox (49.07 ± 4.04) groups (p = 0.001). Moreover, the total collagen ratio in the Trolox group (49.07 ± 4.04) was significantly lower than those in the control group (65.26 ± 7.05) (p = 0.002). There were significant differences in the total muscle ratio between groups. The mean of total muscle ratio in the intact group (49.26 ± 3.36) was significantly higher than those in the control (9.7 ± 4.91) and Trolox (27.46 ± 9.09) groups (p = 0.001). Furthermore, the total muscle ratio was significantly higher in the Trolox group (27.47 ± 9.09) than in the control group (9.7 ± 4.91) (p = 0.001). The collagen and muscle content findings are illustrated in Fig. 2; Table 2.

**Table 2:** Total collagen and muscle rate in the study groups

Variable	Intact group (N = 21)	Control group (N = 21)	Trolox group (N = 21)	P-value
Total collagen (%)	40.11 ± 3.8	65.26 ± 7.05	49.07 ± 4.04	0.001
Total muscle (%)	49.26 ± 3.36	9.11 ± 4.91	27.46 ± 9.09	0.001

### Electromyography

**Fig. 3:**
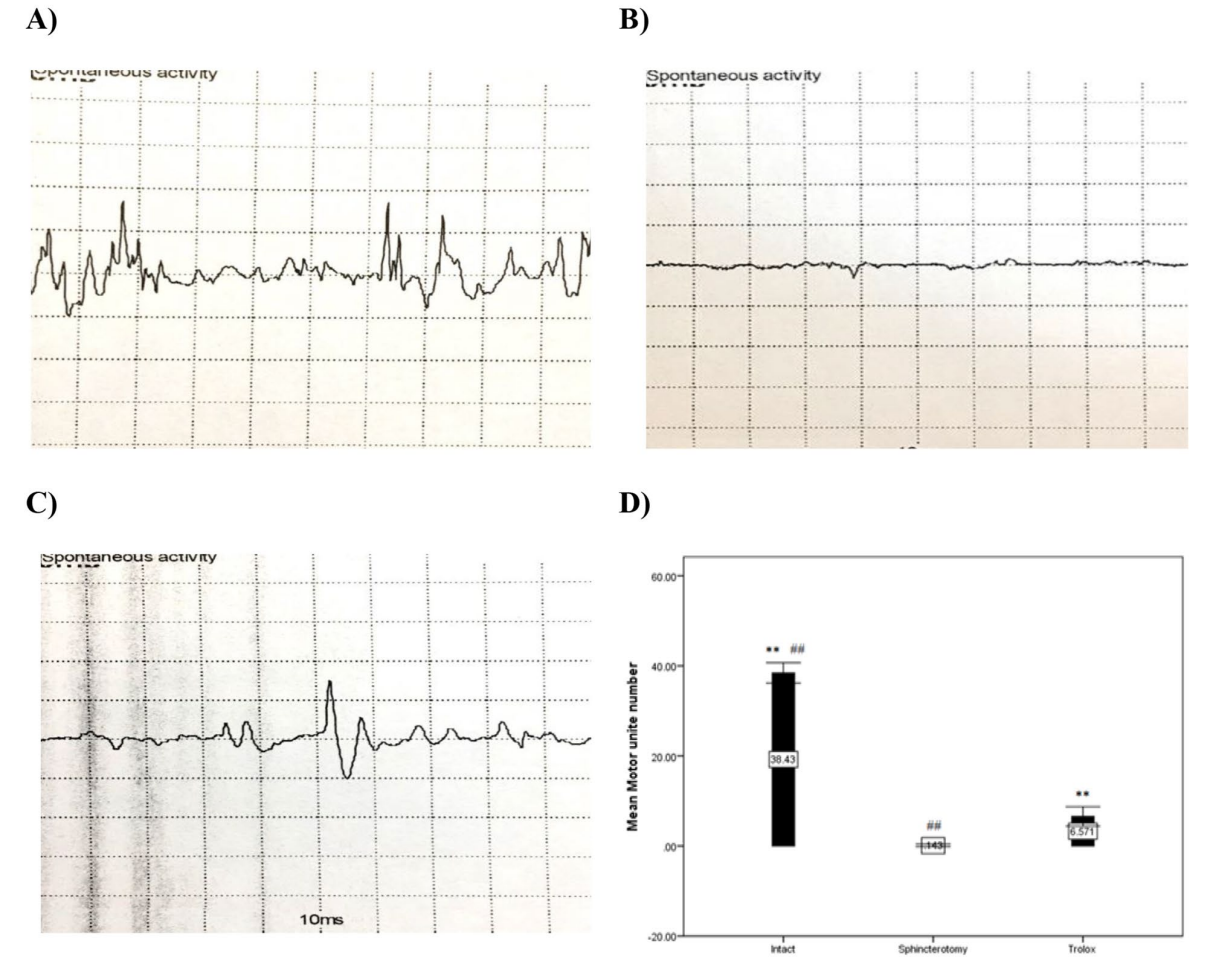
**Motor unite number in the study groups**. (**A**) Intact, (**B**) Control, and (**C**) Trolox group. (**D**) Data are presented as means ± SEM (n = 7 animals per group). **Significant level at p < 0.0001 with the Sphincterotomy group (intact animals). ## Significant level at p < 0.0001 with the Control group

Electromyography determined and evaluated the number of motor units in 21 cases (seven cases for each group). The results demonstrated that the motor unit number was significantly greater in the intact group (38.42 ± 2.99) than in the Trolox (6.58 ± 2.82) and control (0.14 ± 0.38) groups (p = 0.001). In addition, the motor unit number was significantly higher in the Trolox (6.57 ± 2.82) than in the control group (0.14 ± 0.38) (p = 0.001). Electromyography findings are presented in Fig. 3; Table 3.

**Table 3:** Motor unite number in the study groups

Variable	Intact group (N = 7)	Control group (N = 7)	Trolox group (N = 7)	P-value
Motor unite number	38.42 ± 2.99	0.14 ± 0.38	6.57 ± 2.82	0.001

## Discussion

The sphincteroplasty, as the primary treatment option for FI, offers satisfactory results in the short term; however, the FI symptoms could reappear after two years [28, 29] due to fibrous tissue formation in the sphincter defect site, which is not as functional as muscle tissue. Therefore, restoring the muscle tissue in place of fibrous tissue could potentially lead to improved results in the long term. Vitamin E may be an effective strategy for repairing the muscle due to its antioxidant, anti-inflammatory, angiogenic, anti-fibrotic, and protective properties against myocyte loss. Thus, we aimed to evaluate the efficacy of vitamin E in anal sphincter muscle defect of rabbits to establish a treatment approach with long-term and permanent effects.

The anal canal resting pressure is a result of IAS (85%) and EAS (15%) tone, and maximum squeeze pressure is caused by EAS tone [30]. Therefore, a significant reduction in the abovementioned indexes occurs in case of defects in these muscles. In the present study, due to the fourth-degree rupture sphincterotomy (EAS, IAS, and mucosa tear) induction [25] in rabbits, we observed a substantial decline in the resting and maximum squeeze pressures in the sphincterotomy (control) group compared to the intact group. After three months of Trolox administration, there was a significant improvement in the resting pressure of the Trolox group compared to the control group.

α-Tocopherol, the most common vitamin E form in human tissues, protects cells against inflammatory and degenerative processes [31, 32]. Vitamin E has an influential role in muscle repair and protection. In a study by Mancio et al. in 2017, vitamin E supplement administration for 14 days had beneficial effects against myonecrosis, inflammatory response, and oxidative stress [18]. Vitamin E could enhance sarcolemma (muscle cell membrane) integrity, decrease serum creatine kinase level, and inhibit IgG binding to skeletal muscles. Cell membrane repair is the primary mechanism by which vitamin E aids muscle repair [21]. Since vitamin E is fat-soluble, it can penetrate the phospholipid bilayer structure of sarcolemma and influence the physical properties of the cell membrane, such as fluidity, and thus contribute to membrane repair [33].

Moreover, the potent antioxidant effect of vitamin E is another aspect of its role in cell membrane repair, mainly inhibiting lipid peroxidation [34]. In addition to the antioxidant property, the findings of several studies have pointed to the impact of vitamin E on inflammatory factors regulation in different pathological states [35–37]. Vitamin E administration in MDX mice has shown favorable effects on the inflammatory process of muscle and decreases the histological and molecular inflammatory factors that damage muscles [18]. Vitamin E regulates the signal transduction and gene expressions involved in the inflammatory processes [38]. Moreover, α-Tocopherol inhibits NF-κB activity [39], among the main anti-inflammatory properties of vitamin E in injured muscles. Moreover, vitamin E directly decreases the interleukin-6 caused by muscle damage [18]. Therefore, vitamin E has a robust anti-inflammatory effect via sarcolemma protection, decreasing lipid peroxidation, inhibiting NF-κB, and reducing interleukin-6 levels.

Anti-fibrosis is another critical function of vitamin E, achieved through TGF-β expression regulation [22]. TGF- β is a profibrogenic cytokine that is increased in fibrotic lesions and scar formation up to four times. Accordingly, the present study’s findings demonstrate the significant collagen decline in the sphincterotomy site in the Trolox group relative to the control group. In general, the healing of sphincter tissue occurs through several main mechanisms that vitamin E provides. These mechanisms include anti-fibrotic, anti-inflammatory, antioxidant properties and protection of myocytes against inflammatory and apoptotic processes of damaged sphincter tissue. Reducing fibrosis tissue on one hand and preventing the loss of sphincter tissue myocytes along with muscle regeneration on the other hand increases the ratio of muscle tissue to fibrosis tissue in the lesion site. In other words, maintaining the myocytes of the sphincter tissue is a far more important factor than the production of new myocytes in achieving the desired results regarding sphincter muscle tissue function.

Increases the ratio of muscle tissue to fibrosis tissue in the lesion site is proved to be a crucial factor in improving anal sphincter injuries and FI, according to animal studies and clinical trials. In 2020, Sarveazad et al. revealed that the significant decrease in collagen content secondary to human adipose-derived stem cells and low-level laser therapy in the rabbit’s anal sphincterotomy site resulted in muscle content increase compared to the control group and a rise in the resting and maximum squeeze pressures of anal sphincter tone and motor units’ number in electromyography. Our findings were similar regarding the collagen content reduction, increase in the anal sphincter tone in resting and maximum squeeze, and electromyography data [26]. The results of a clinical trial by Sarveazad et al., published in 2017, showed that the increases the ratio of muscle tissue to fibrosis tissue at the sphincterotomy site of patients who had FI due to EAS injury improved the Wexner score (FI score) and significantly increased the number of motor units [27].

The rise in the resting and maximum squeeze pressures of anal sphincter tone is caused by the increases the ratio of muscle tissue to fibrosis tissue of the sphincterotomy site. In 2014, Baldelli et al. showed that Trolox enhances myogenesis by regulating proliferative-activated receptor gamma coactivator-1 alpha (PGC-1α) [40].

The strength of the present study includes long follow-up time (90 days) and performing functional tests. Nevertheless, our study has some limitations. We did not evaluate the molecular pathways involved in myogenesis, and the study design was not dose-dependent. Thus, it is recommended to design further studies evaluating different doses of Trolox and investigate the involved molecular pathways in addition to the functional tests and histological evaluation.

Because no patient with sphincter lesions knows before being injured, in order to mimic the most similarity with clinical injuries, it is suggested that future studies be designed in such a way that Trolox is administered after sphincterotomy (not at the time of sphincterotomy or before). Among other suggestions, in future studies, Trolox should be administered in different degrees of sphincter damage (with different lengths and extension) so that the real effectiveness of Trolox in repairing the sphincter muscle can be judged.

## Conclusion

In conclusion, Trolox administration in rabbit sphincterotomy models can decrease collagen and increase muscle content in the EAS defect location. Moreover, the increase in muscle content improves electromyography findings and increases the anal sphincter tone. According to these results, Trolox can be a potential treatment option in patients with FI.

## Data Availability

The datasets generated during and/or analyzed during the current study are available from the corresponding author on reasonable request.

## List of Abbreviations

PFD: Pelvic Floor Disorder
FI: Fecal Incontinence
EAS: External Anal Sphincter
IAS: Internal Anal Sphincter
MUAP: Motor Unit Action Potential
PGC-1α: Proliferative-Activated Receptor Gamma Coactivator-1 lpha (PGC-1α)
